# Tumor apparent diffusion coefficient combined with the lipid-to-creatine ratio for preoperative differentiation of primary central nervous system lymphoma from high-grade glioma: a single-center diagnostic model development and internal validation study

**DOI:** 10.3389/fonc.2026.1941153

**Published:** 2026-09-15

**Authors:** Lei Geng, Subo Zhang, Zhaoting Zhang, Lei Xu, Xuzhu Gao

**Affiliations:** 1 Department of Medical Imaging, The Second People’s Hospital of Lianyungang (The Oncology Hospital of Lianyungang), Affiliated Lianyungang Clinical College of Nantong University, and The Second People's Hospital of Lianyungang Affiliated to Kangda College of Nanjing Medical University, Lianyungang, Jiangsu, China; 2 Department of Neurology, The Second People’s Hospital of Lianyungang, Lianyungang, Jiangsu, China; 3 Central Laboratory, The Second People’s Hospital of Lianyungang, Lianyungang, Jiangsu, China

**Keywords:** apparent diffusion coefficient, high-grade glioma, internal validation, lipid-to-creatine ratio, magnetic resonance spectroscopy, primary central nervous system lymphoma

## Abstract

**Background and purpose:**

Primary central nervous system lymphoma (PCNSL) and high-grade glioma (HGG) may share overlapping conventional MRI appearances. We developed and internally validated a parsimonious model combining tumor apparent diffusion coefficient (ADC) and the lipid-to-creatine ratio (Lip/Cr), compared it with every single predictor, and evaluated the secondary contribution of proximal peritumoral ADC.

**Materials and methods:**

This retrospective, availability-based cohort included 83 consecutive eligible patients with pathologically confirmed PCNSL (n = 38) or HGG (n = 45) who underwent pretreatment 3.0-T MRI with diffusion-weighted imaging and proton magnetic resonance spectroscopy. Four prespecified logistic models were evaluated. Internal validation used 2,000 stratified bootstrap resamples with standardization repeated within each resample. Distribution-appropriate summaries, paired incremental performance, empirical calibration, sensitivity analyses, and exploratory decision-curve analysis were reported.

**Results:**

Tumor ADC was lower and Lip/Cr was higher in PCNSL than in HGG (both P < 0.001). The tumor ADC plus Lip/Cr model achieved an apparent AUC of 0.925 and an optimism-corrected AUC of 0.918 (95% CI, 0.868–0.980); the corrected Brier score was 0.114 (95% CI, 0.073–0.160). Relative to Lip/Cr alone, the corrected changes were small and uncertain (ΔAUC, 0.021; 95% CI, −0.039 to 0.055; ΔBrier, −0.020; 95% CI, −0.044 to 0.023). Adding proximal peritumoral ADC also produced small and uncertain changes (ΔAUC, 0.004; 95% CI, −0.039 to 0.023; ΔBrier, −0.005; 95% CI, −0.018 to 0.027). Findings were directionally stable after log transformation of Lip/Cr and restriction of the HGG comparator to IDH-wildtype glioblastoma.

**Conclusions:**

Tumor ADC plus Lip/Cr showed favorable exploratory internal-validation performance, but superiority over Lip/Cr alone was not established. The model requires multicenter external validation with prespecified MRS quality control and should support, rather than replace, expert interpretation and histopathologic confirmation.

## Highlights

- Tumor ADC and Lip/Cr provide complementary diffusion and metabolic information, but the incremental advantage over Lip/Cr alone was small and uncertain.
- The primary model had an optimism-corrected AUC of 0.918 and a corrected Brier score of 0.114.
- Proximal peritumoral ADC remained a prespecified secondary extension because its incremental performance was small and imprecise.

## 1 Introduction

Primary central nervous system lymphoma (PCNSL) is an aggressive extranodal lymphoma confined to the central nervous system; the majority of cases are diffuse large B-cell lymphomas. High-grade gliomas (HGGs) comprise molecularly heterogeneous WHO grade 3 and 4 diffuse gliomas. Treatment pathways differ substantially: HGG is generally managed with maximal safe resection followed by molecularly informed adjuvant treatment, whereas PCNSL usually requires tissue confirmation followed by high-dose methotrexate–based therapy. Premature corticosteroid administration can reduce biopsy yield in suspected PCNSL, but imaging alone cannot determine the treatment approach (1–4, 16, 17, 22).

Conventional MRI findings overlap considerably. Although homogeneous enhancement and marked diffusion restriction favor PCNSL, necrosis, ring enhancement, hemorrhage, multifocality, and extensive edema may occur in either entity. Tumor apparent diffusion coefficient (ADC) is generally lower in PCNSL than in HGG, but overlap remains (5–8). Proton magnetic resonance spectroscopy (MRS) provides complementary metabolic information; however, lipid-region measurements are susceptible to voxel placement, extracranial lipid contamination, baseline instability, spectral broadening, and peak-fitting variability (9–11).

Many studies emphasize the highest apparent area under the receiver operating characteristic curve (AUC) or use data-driven variable selection in small samples. We therefore prespecified low-dimensional logistic models and repeated preprocessing within bootstrap validation. The primary objective was to internally validate tumor ADC plus Lip/Cr and quantify its incremental value relative to both tumor ADC alone and Lip/Cr alone. The secondary objective was to estimate the incremental contribution of proximal peritumoral ADC. Because the HGG comparator included several molecular entities, a subtype-restricted sensitivity analysis was also performed.

## 2 Materials and methods

### 2.1 Study design and participants

This retrospective single-center diagnostic model development study used an availability-based cohort of patients examined between January 2021 and December 2025 at The Second People’s Hospital of Lianyungang. Consecutive eligible patients had histopathologically confirmed PCNSL or WHO grade 3–4 diffuse glioma and pretreatment 3.0-T MRI, including CE-T1WI, T2-FLAIR, DWI/ADC, and proton MRS. Exclusion criteria were prior surgery, radiotherapy, chemotherapy, or corticosteroid treatment before MRI; missing key sequences; severe artifacts or inadequate spectral quality precluding quantitative assessment; or incomplete pathologic classification. The de-identified analytic file contained 83 complete cases (38 PCNSL and 45 HGG). Candidate-level counts excluded before analytic-file assembly were not retained; this screening-log limitation is shown explicitly in **Figure 1** rather than reconstructed retrospectively.

**Figure 1:**
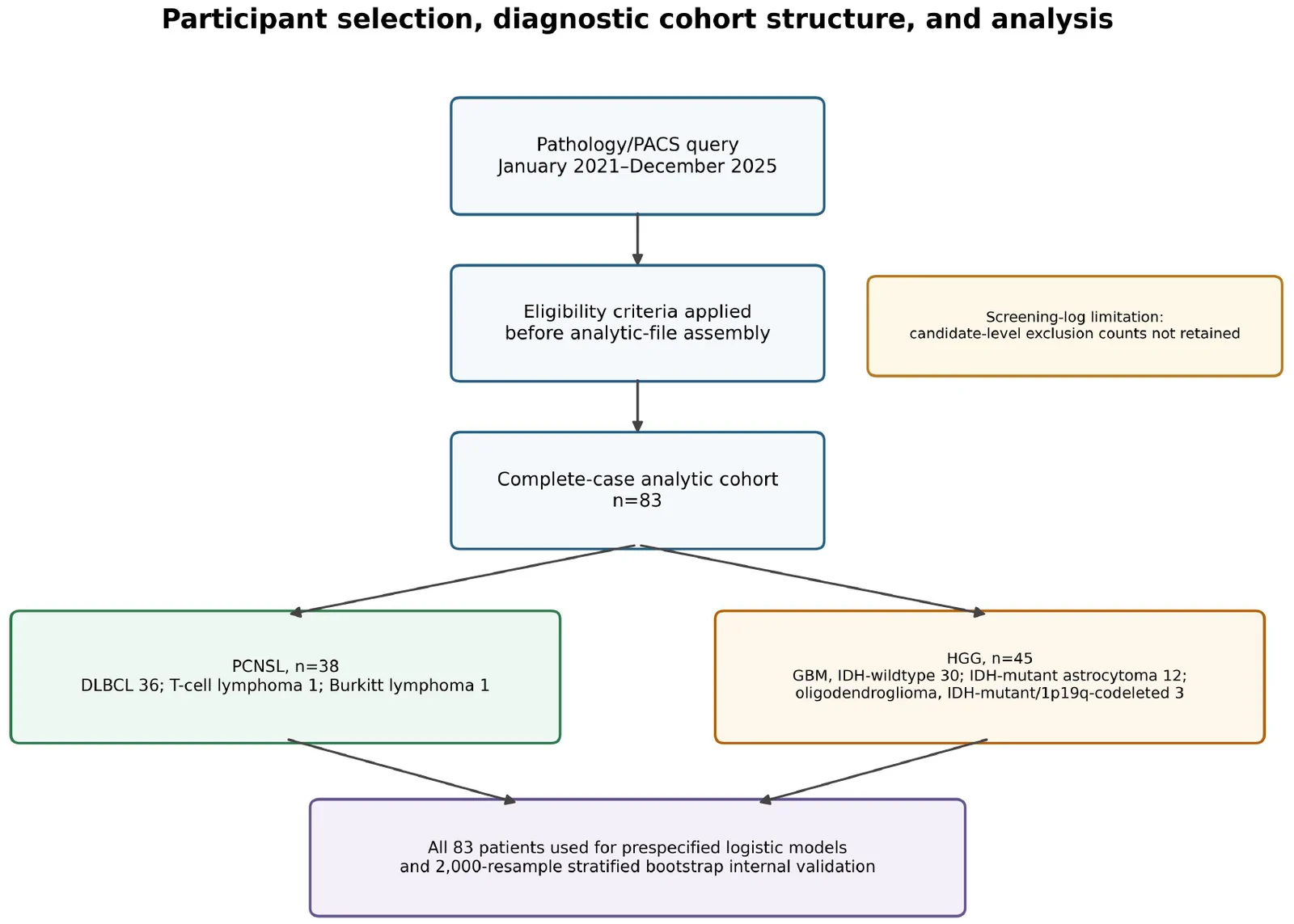
Participant selection, diagnostic cohort structure, and analysis. Candidate-level exclusion counts before assembly of the de-identified analytic file were unavailable and are explicitly identified as a limitation.

The institutional ethics committee approved the study (approval no. 2024K047) and waived written informed consent because of the retrospective design. The study involved human participants and was conducted in accordance with the Declaration of Helsinki. Reporting was guided by STARD 2015 and TRIPOD+AI (13, 20).

### 2.2 Reference standard and pathologic classification

Histopathology from stereotactic biopsy or surgical tissue was the reference standard. Diagnoses were assigned based on the contemporaneous pathology report and molecular work-up according to applicable WHO criteria. PCNSL comprised diffuse large B-cell lymphoma (DLBCL) (n = 36), T-cell lymphoma (n = 1), and Burkitt lymphoma (n = 1). HGG comprised glioblastoma, isocitrate dehydrogenase (IDH)-wildtype, WHO grade 4 (n = 30); astrocytoma, IDH-mutant, WHO grade 4 (n = 7); astrocytoma, IDH-mutant, WHO grade 3 (n = 5); and oligodendroglioma, IDH-mutant and 1p/19q-codeleted, WHO grade 3 (n = 3). Because these entities differ biologically, the pooled HGG analysis was treated as the primary pragmatic comparison, and PCNSL versus IDH-wildtype glioblastoma was evaluated as a sensitivity analysis.

### 2.3 MRI and MRS acquisition

MRI was performed on a Siemens 3.0-T system with a 20-channel head coil. Axial T2-FLAIR parameters were repetition time/echo time/inversion time, 9,000/89/2,500 ms; section thickness, 5 mm; intersection gap, 1 mm; field of view, 230 × 230 mm; and matrix, 256 × 256. DWI used spin-echo echo-planar imaging with repetition time/echo time, 5,200/80 ms; b values, 0 and 1,000 s/mm²; section thickness, 5 mm; intersection gap, 1 mm; field of view, 230 × 230 mm; and matrix, 128 × 128. ADC maps were generated automatically. CE-T1WI used three-dimensional MPRAGE after administration of 0.1 mmol/kg gadolinium-based contrast material (repetition time/echo time/inversion time, 1,900/2.5/900 ms; flip angle, 9°; section thickness, 1 mm; field of view, 256 × 256 mm; matrix, 256 × 256).

Two-dimensional multivoxel proton MRS used point-resolved spectroscopy (repetition time/echo time, 1,500/144 ms; nominal voxel size, approximately 10 × 10 × 15 mm). The volume of interest covered the enhancing solid tumor and avoided necrosis, hemorrhage, bone, subcutaneous fat, and cerebrospinal fluid. The reported Lip/Cr was the workstation-derived peak-area ratio of the lipid-dominant 0.9–1.3-ppm envelope to creatine at approximately 3.03 ppm. At TE 144 ms, lipid and lactate contributions in the 1.3-ppm region may overlap; therefore, Lip/Cr is an operational workstation label rather than proof of a chemically pure lipid signal. Spectra required a stable baseline, adequate water suppression, identifiable creatine and major metabolites, and no dominant extracranial lipid contamination. Cho/Cr and Cho/NAA were obtained from the same selected voxel and automated fit. Numeric signal-to-noise ratio, full width at half maximum, and repeated-reader MRS measurements were not retained, so MRS reproducibility could not be quantified retrospectively.

### 2.4 ADC image analysis

Two neuroradiologists with more than 5 years of experience independently measured ADC while blinded to histopathology. CE-T1WI and T2-FLAIR were co-displayed with ADC maps on the workstation and visually aligned using anatomic landmarks; no deformable image registration was performed. Cases with severe echo-planar distortion or irreconcilable section mismatch were excluded during eligibility assessment. The enhancing solid-tumor boundary was localized on CE-T1WI and transferred to the ADC map. A circular or oval ROI was placed within the visually lowest-ADC viable enhancing portion while avoiding necrosis, hemorrhage, cystic change, vessels, and cerebrospinal fluid. The reported value was the mean ADC within each ROI, not the minimum single-voxel ADC. The index section containing the largest viable enhancing area and the immediately adjacent superior and inferior sections were measured and averaged. The available worksheet did not retain ROI area; consequently, a retrospective ROI-size or whole-tumor-ADC sensitivity analysis was not possible.

Proximal peritumoral ROIs were centered 0–10 mm outside the enhancing margin within contiguous T2-FLAIR hyperintensity. Distal ROIs were centered more than 10 mm outside the enhancing margin but within the same contiguous abnormality. For each patient, readers attempted to maintain similar ROI geometry across the three sections and repositioned ROIs to account for visible distortion or partial-volume effects. Contralateral normal-appearing white matter was sampled in a mirrored or anatomically comparable region. ROIs were mutually exclusive. For multifocal disease, the largest lesion with complete MRS coverage was selected. The mean of the measurements from both readers was used for modeling. Relative ADC was calculated as tumor ADC divided by contralateral white-matter ADC; it was excluded from candidate models because it is algebraically dependent on tumor ADC, adds measurement noise from the reference ROI, and would be redundant in this small dataset.

### 2.5 Model specification, sample size, and internal validation

The outcome was PCNSL = 1 and HGG = 0. Four ordinary binary logistic models were prespecified: tumor ADC; Lip/Cr; tumor ADC plus Lip/Cr (primary candidate); and the primary model plus proximal peritumoral ADC (secondary extension). No univariable screening, stepwise selection, or automated feature selection was used. No *a priori* sample-size calculation was performed; sample size was determined by all eligible cases available during the study period. The 38 PCNSL events corresponded to approximately 19 events per predictor for the two-parameter model and 12.7 events per predictor for the three-parameter model, but these ratios do not guarantee stable calibration or small incremental estimates. Accordingly, all analyses are exploratory and require external validation.

All 83 patients were used for model fitting. Internal validation used 2,000 stratified bootstrap resamples with random seed 20260715. For each resample, predictor means and population standard deviations were re-estimated, the model was refitted, and performance was evaluated in both the resample and the original cohort. Optimism was the resample performance minus test-on-original performance; this was subtracted from apparent performance. The 95% percentile confidence intervals were calculated from the distribution of optimism-adjusted estimates. Paired model differences used the same stratified resamples. AUC, Brier score, calibration intercept, and calibration slope were reported, consistent with transparent prediction-model guidance (12–14). **Figure 2** shows only apparent development-sample ROC curves; corrected estimates are in **Table 1**.

**Figure 2:**
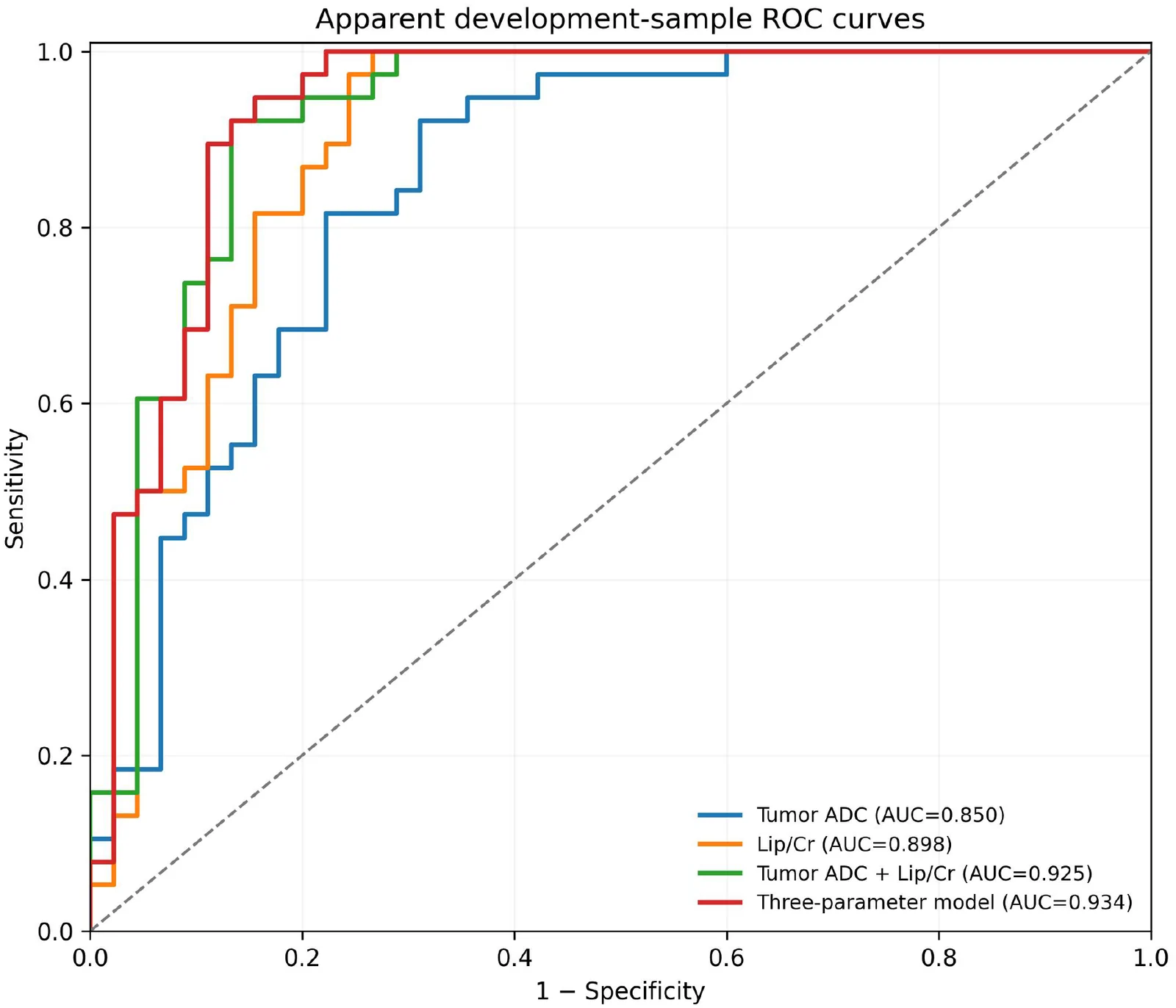
Apparent development-sample receiver operating characteristic curves for the four prespecified models. Optimism-corrected estimates are reported in **Table 1**.

**Table 1:** Apparent and optimism-corrected model performance.

Model	Apparent AUC	Corrected AUC (95% CI)	Apparent brier	Corrected brier (95% CI)	Corrected intercept/slope
Tumor ADC	0.850	0.851 (0.778–0.939)	0.157	0.161 (0.122–0.208)	0.005/0.999
Lip/Cr	0.898	0.897 (0.837–0.971)	0.130	0.134 (0.095–0.180)	0.005/0.988
Tumor ADC + Lip/Cr	0.925	0.918 (0.868–0.980)	0.105	0.114 (0.073–0.160)	–0.020/0.904
Three-parameter model	0.934	0.922 (0.874–0.979)	0.096	0.109 (0.065–0.160)	–0.003/0.836

Corrected values were obtained from 2,000 stratified bootstrap resamples with preprocessing repeated within each resample.

### 2.6 Statistical and sensitivity analyses

Normality was assessed separately within diagnostic groups using Shapiro–Wilk tests and distribution plots. Variables compatible with normality in both groups were summarized as mean ± standard deviation and compared using Welch’s t-test. Right-skewed metabolite ratios (Cho/Cr, Cho/NAA, and Lip/Cr) were summarized as median (interquartile range) and compared using the Mann–Whitney U test. Categorical variables were compared using chi-square tests or Fisher exact tests. The final analytic dataset had no missing values for prespecified predictors, so complete-case analysis included all 83 patients; exclusions for inadequate MRS quality occurred before analytic-file assembly and cannot be quantified from the retained dataset.

Interobserver agreement for ADC was assessed using a two-way random-effects, absolute-agreement, single-measure ICC(2,1). Bland–Altman analysis reported the rounded mean difference and 95% limits of agreement; linear regression of reader difference on reader mean assessed proportional bias. Standardized logistic coefficients, standard errors, odds ratios, and 95% confidence intervals were reported for both the primary and extended models. Sensitivity analyses used log-transformed Lip/Cr and restriction to PCNSL versus IDH-wildtype glioblastoma. Empirical calibration was displayed in five equal-frequency groups with Wilson 95% confidence intervals. Threshold performance was shown at 0.30, 0.50, and 0.70; these thresholds are descriptive, not clinical action thresholds. Exploratory apparent decision-curve analysis assessed net benefit from 0.10 to 0.80 (21). Two-sided P < 0.05 was used for conventional comparisons; incremental performance was interpreted based on magnitude and uncertainty rather than significance thresholds.

## 3 Results

### 3.1 Cohort and quantitative MRI findings

The complete-case cohort included 38 patients with PCNSL and 45 with HGG. All prespecified predictor fields were complete in the retained analytic dataset. Age and sex distributions were similar. Tumor ADC and relative ADC were lower in PCNSL, whereas proximal peritumoral ADC and Lip/Cr were higher. Cho/Cr, Cho/NAA, and Lip/Cr were non-normally distributed and were therefore reported as median (interquartile range). Distal peritumoral ADC, contralateral white-matter ADC, Cho/Cr, and Cho/NAA did not differ significantly (**Table 2**; **Supplementary Figure S1**).

**Table 2:** Demographic and quantitative MRI characteristics.

Variable	PCNSL (n = 38)	HGG (n = 45)	P value
Age, years	63.21 ± 9.97	64.71 ± 9.78	0.493
Sex, male/female	18/20	22/23	0.890
Tumor ADC, ×10^−3^ mm²/s	0.76 ± 0.13	1.00 ± 0.19	<0.001
Proximal peritumoral ADC, ×10^−3^ mm²/s	1.59 ± 0.15	1.42 ± 0.19	<0.001
Distal peritumoral ADC, ×10^−3^ mm²/s	1.48 ± 0.17	1.50 ± 0.17	0.601
Contralateral white-matter ADC, ×10^−3^ mm²/s	0.85 ± 0.03	0.84 ± 0.03	0.150
Cho/Cr, median (IQR)	1.97 (1.63–2.40)	1.66 (1.49–2.12)	0.127
Cho/NAA, median (IQR)	1.83 (1.34–2.38)	1.71 (1.38–2.48)	0.728
Lip/Cr, median (IQR)	5.76 (4.53–7.19)	2.57 (1.41–3.78)	<0.001
Relative ADC	0.90 ± 0.15	1.19 ± 0.21	<0.001

Normally distributed variables are presented as mean ± SD and were compared with Welch’s t-test. Cho/Cr, Cho/NAA, and Lip/Cr are presented as median (IQR) and were compared with the Mann–Whitney U test. Sex was compared with the chi-square test. IQR, interquartile range.

Interobserver agreement was excellent for all ADC measurements (ICC > 0.90; **Table 3**). For tumor ADC, the Bland–Altman mean reader difference rounded to 0.000 × 10^−3^ mm²/s, with 95% limits of agreement from −0.110 to 0.110 × 10^−3^ mm²/s. There was no evidence of proportional bias (slope, −0.034, P = 0.278; **Figure 3**).

**Table 3:** Interobserver agreement for ADC measurements.

Measurement	ICC(2,1)	95% CI
Tumor ADC	0.962	0.927–0.987
Proximal peritumoral ADC	0.948	0.913–0.973
Distal peritumoral ADC	0.932	0.897–0.957
Contralateral white-matter ADC	0.908	0.853–0.933

ICC(2,1), two-way random-effects, absolute-agreement, single-measure intraclass correlation coefficient.

**Figure 3:**
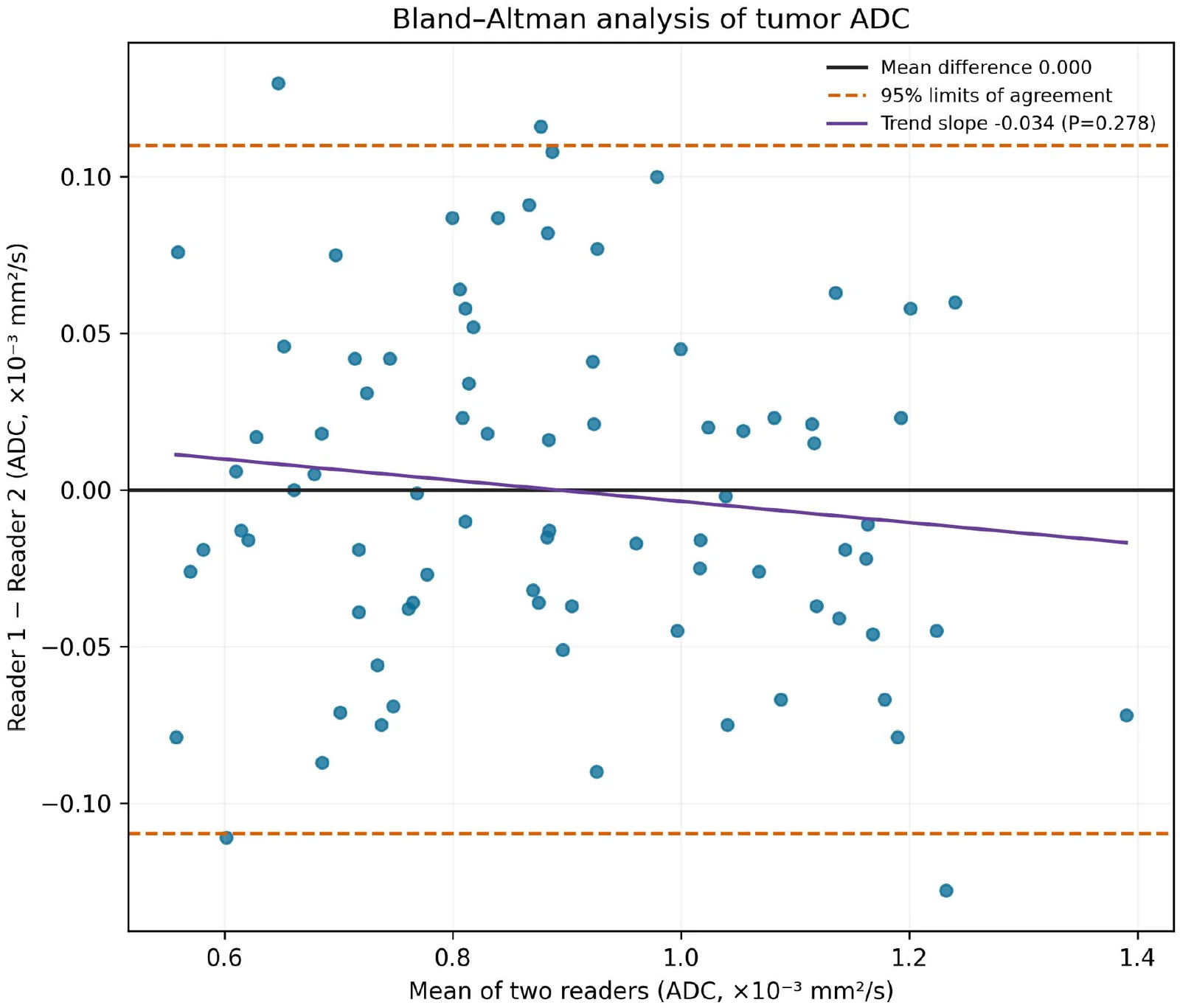
Bland–Altman plot for tumor ADC measured by two readers. The rounded mean difference was 0.000 × 10^−3^ mm²/s, and the 95% limits of agreement were −0.110 to 0.110 × 10^−3^ mm²/s. The regression line assesses proportional bias (slope, −0.034; P = 0.278).

### 3.2 Logistic regression coefficients

In the primary model, lower standardized tumor ADC (OR, 0.263; 95% CI, 0.117–0.591) and higher standardized Lip/Cr (OR, 6.215; 95% CI, 2.215–17.436) were associated with PCNSL. In the prespecified extended model, the proximal peritumoral ADC estimate was positive but imprecise (OR, 2.326; 95% CI, 0.971–5.571). Its status as a secondary extension was determined by the prespecified model hierarchy, not by whether the P value was just above or below 0.05 (**Table 4**).

**Table 4:** Standardized logistic regression coefficients for primary and extended models.

Model	Variable	β	SE	P	OR	95% CI
Tumor ADC + Lip/Cr	Intercept	–0.356	0.351	0.311	0.701	0.352–1.394
	Tumor ADC	–1.336	0.414	0.001	0.263	0.117–0.591
	Lip/Cr	1.827	0.526	<0.001	6.215	2.215–17.436
Three-parameter	Intercept	–0.431	0.370	0.244	0.650	0.315–1.342
	Tumor ADC	–1.263	0.447	0.005	0.283	0.118–0.680
	Lip/Cr	1.672	0.550	0.002	5.322	1.809–15.650
	Proximal peritumoral ADC	0.844	0.446	0.058	2.326	0.971–5.571

ORs correspond to a one-population-standard-deviation increase in the predictor. PCNSL = 1 and HGG = 0.

### 3.3 Model performance, calibration, and incremental value

The apparent AUCs of tumor ADC alone and Lip/Cr alone were 0.850 and 0.898. The primary model had an apparent AUC of 0.925 and an optimism-corrected AUC of 0.918 (95% CI, 0.868–0.980). Its corrected Brier score was 0.114 (95% CI, 0.073–0.160), its corrected calibration intercept was −0.021, and its corrected slope 0.903. The slope confidence interval was wide (0.451–1.341), indicating limited calibration precision. **Figure 2** presents apparent ROC curves only; **Figure 4** presents empirical apparent calibration in equal-frequency groups with Wilson intervals.

**Figure 4:**
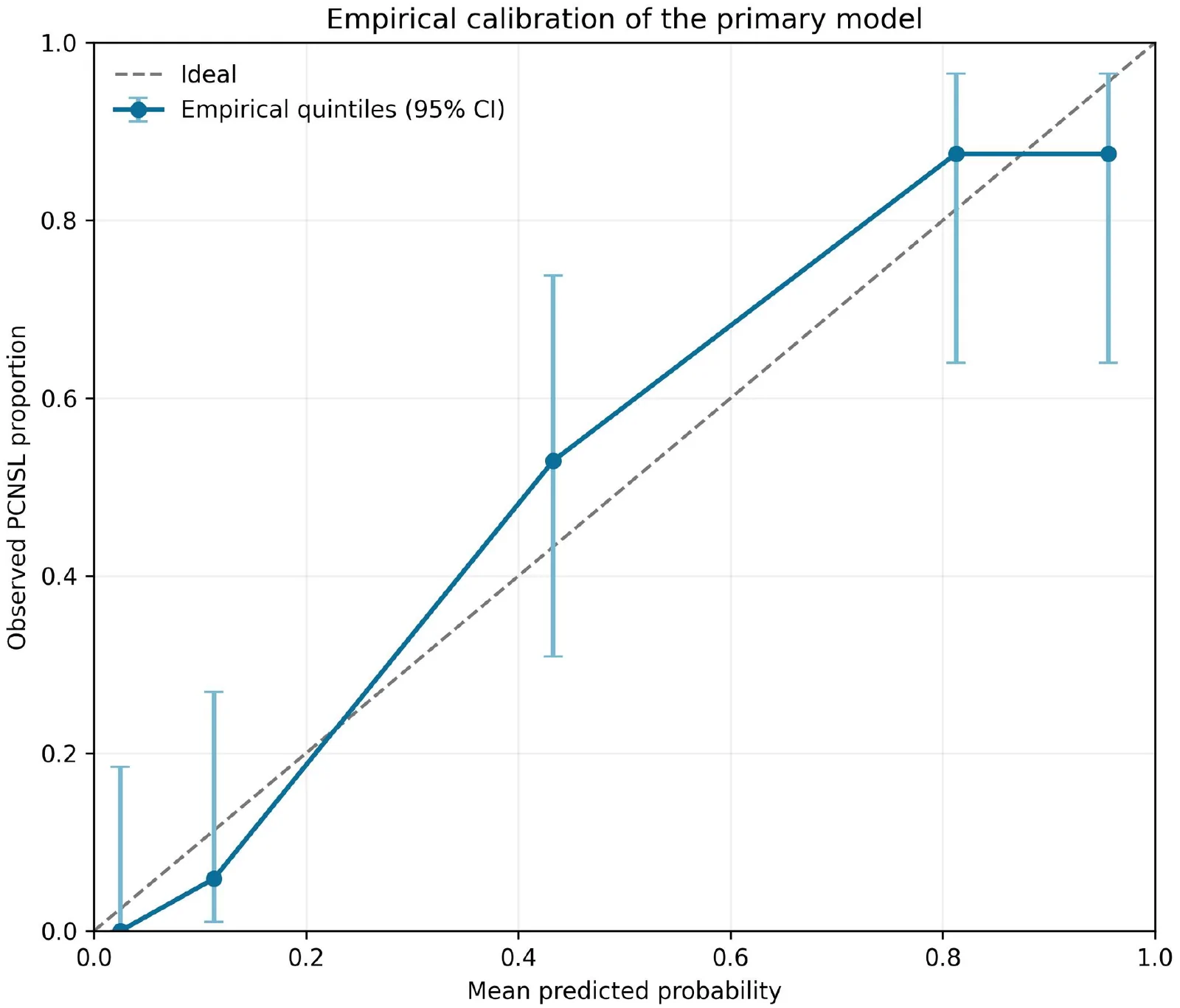
Empirical apparent calibration of the primary model in five equal-frequency groups. Points show the mean predicted probability and observed PCNSL proportion; error bars show Wilson 95% confidence intervals. Optimism-corrected intercept and slope are reported in **Table 1**.

Relative to tumor ADC alone, the primary model improved the corrected AUC by 0.067 and reduced the corrected Brier score by 0.047. However, comparison with the strongest single predictor, Lip/Cr, showed much smaller and uncertain gains: ΔAUC 0.021 and ΔBrier −0.020, with both confidence intervals crossing zero. Adding proximal peritumoral ADC yielded similarly small and uncertain changes. Therefore, the data support complementary information but do not establish superiority of the combined model over Lip/Cr alone (**Table 5**).

**Table 5:** Paired incremental performance and sensitivity analyses.

Analysis	AUC result	Brier result	Interpretation
Primary vs tumor ADC	0.067 (–0.016 to 0.118)	–0.047 (–0.075 to –0.003)	Paired bootstrap comparison
Primary vs Lip/Cr	0.021 (–0.039 to 0.055)	–0.020 (–0.044 to 0.023)	Paired bootstrap comparison
Extended vs primary	0.004 (–0.039 to 0.023)	–0.005 (–0.018 to 0.027)	Paired bootstrap comparison
Log-transformed Lip/Cr model	Corrected AUC 0.927 (0.880–0.985)	Corrected Brier 0.105 (0.065–0.150)	Distributional sensitivity
PCNSL vs IDH-wildtype GBM (n = 68)	Corrected AUC 0.891 (0.821–0.977)	Corrected Brier 0.120 (0.071–0.172)	Subtype-restricted sensitivity

Δ values are the first model minus the comparator. Negative ΔBrier favors the first model. Sensitivity analyses used 2,000 bootstrap resamples.

Sensitivity analyses were directionally consistent. Log-transforming Lip/Cr yielded a corrected AUC of 0.927 and a corrected Brier score of 0.105. Restricting the comparator to IDH-wildtype glioblastoma (38 PCNSL and 30 GBM) yielded a corrected AUC of 0.891 and a corrected Brier score of 0.120. The latter estimate was lower and imprecise, reinforcing the need for larger subtype-specific validation rather than assuming that pooled HGG performance applies uniformly.

### 3.4 Threshold analyses and exploratory clinical utility

At thresholds of 0.30, 0.50, and 0.70, sensitivity decreased from 0.947 to 0.737, while specificity increased from 0.800 to 0.911 (**Table 6**). These thresholds were selected only to illustrate trade-offs. Apparent exploratory decision-curve analysis showed positive net benefit for the primary model across much of the 0.10–0.80 range (**Figure 5**), but the curve was not externally validated, and no clinical action threshold or treatment consequence was prespecified. It therefore cannot establish clinical utility.

**Table 6:** Apparent classification performance at descriptive probability thresholds.

Threshold	Sensitivity	Specificity	PPV	NPV	Accuracy	TP/FP/TN/FN
0.30	0.947	0.800	0.800	0.947	0.867	36/9/36/2
0.50	0.868	0.867	0.846	0.886	0.867	33/6/39/5
0.70	0.737	0.911	0.875	0.804	0.831	28/4/41/10

PPV and NPV depend on prevalence. Thresholds were not defined as clinical action thresholds.

**Figure 5:**
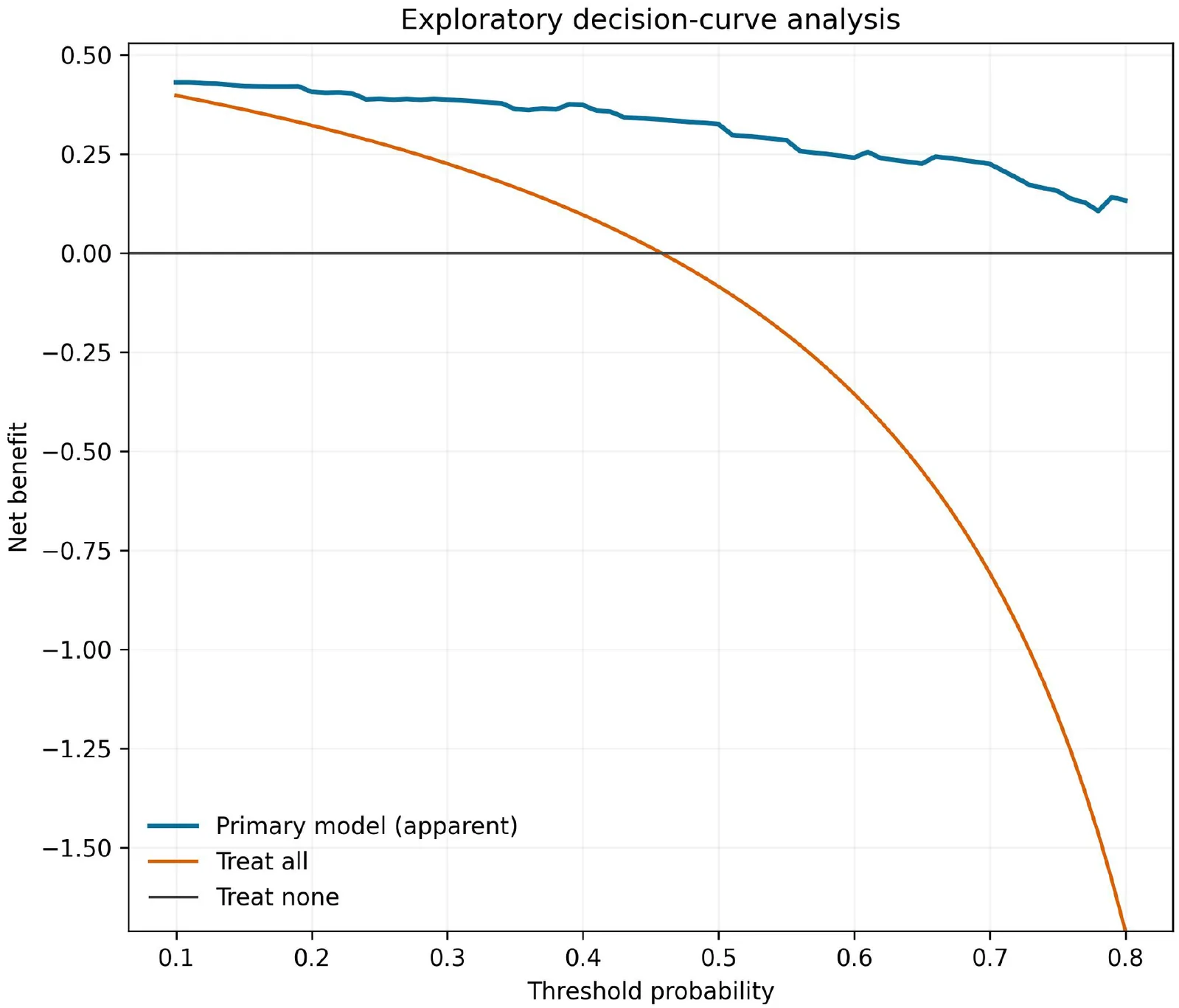
Exploratory apparent decision-curve analysis of the primary model. The plot is hypothesis-generating because no clinical action threshold was prespecified and no external validation or reader-impact study was performed.

### 3.5 Model equation

The standardized primary model was: logit(PPCNSL) = −0.356 − 1.336 × Z(tumor ADC) + 1.827 × Z(Lip/Cr). Means and population standard deviations were 0.890 and 0.199, respectively, for tumor ADC and 4.336 and 2.612, respectively, for Lip/Cr. In original units: logit(PPCNSL) = 2.589 − 6.715 × tumor ADC + 0.699 × Lip/Cr. The extended equation was: logit(PPCNSL) = −4.176 − 6.348 × tumor ADC + 0.640 × Lip/Cr + 4.420 × proximal peritumoral ADC. The equations are provided for research replication and external validation, not as treatment thresholds.

### 3.6 Illustrative cases

**Figure 6** shows an atypical HGG with deep and subcortical involvement, heterogeneous enhancement, and heterogeneous diffusion restriction. Tumor ADC was 1.14 × 10^−3^ mm²/s, proximal peritumoral ADC was 1.56 × 10^−3^ mm²/s, and Lip/Cr was 2.60. The combined model favored HGG, consistent with histopathology.

**Figure 6:**
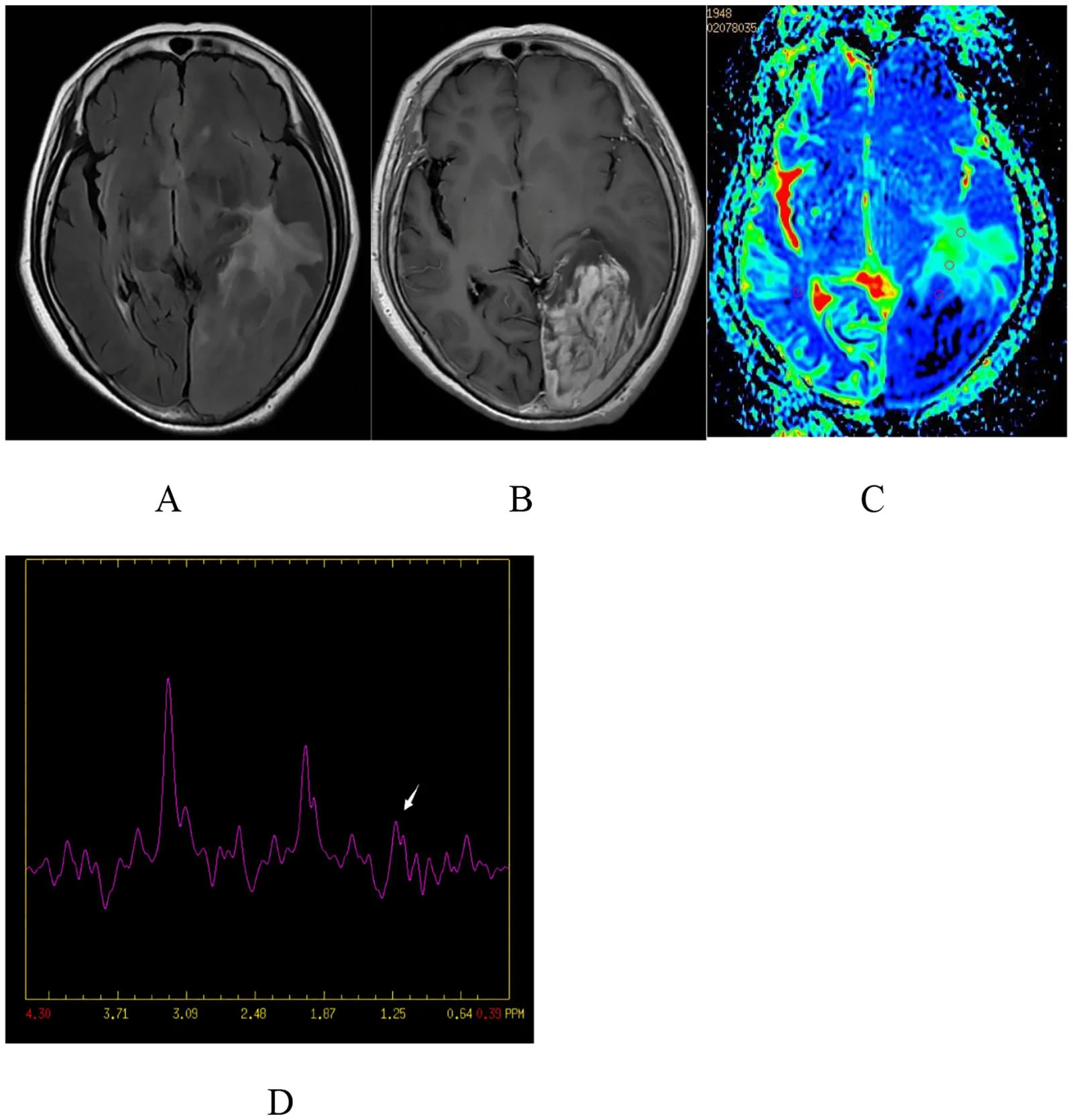
A 50-year-old man with atypical high-grade glioma in the left temporo-occipital lobe. **(A)** T2-FLAIR demonstrates heterogeneous hyperintensity with peritumoral edema. **(B)** Contrast-enhanced T1-weighted imaging shows irregular heterogeneous enhancement. **(C)** ADC map demonstrates heterogeneous diffusion restriction. **(D)** Proton MRS shows elevated choline, reduced N-acetylaspartate, and lipid/lactate peaks.

**Figure 7** shows an atypical PCNSL with necrosis and predominantly ring-like enhancement. Tumor ADC was 0.65 × 10^−3^ mm²/s, proximal peritumoral ADC was 1.73 × 10^−3^ mm²/s, and Lip/Cr was 7.70. The combined model favored PCNSL, which was confirmed histopathologically.

**Figure 7:**
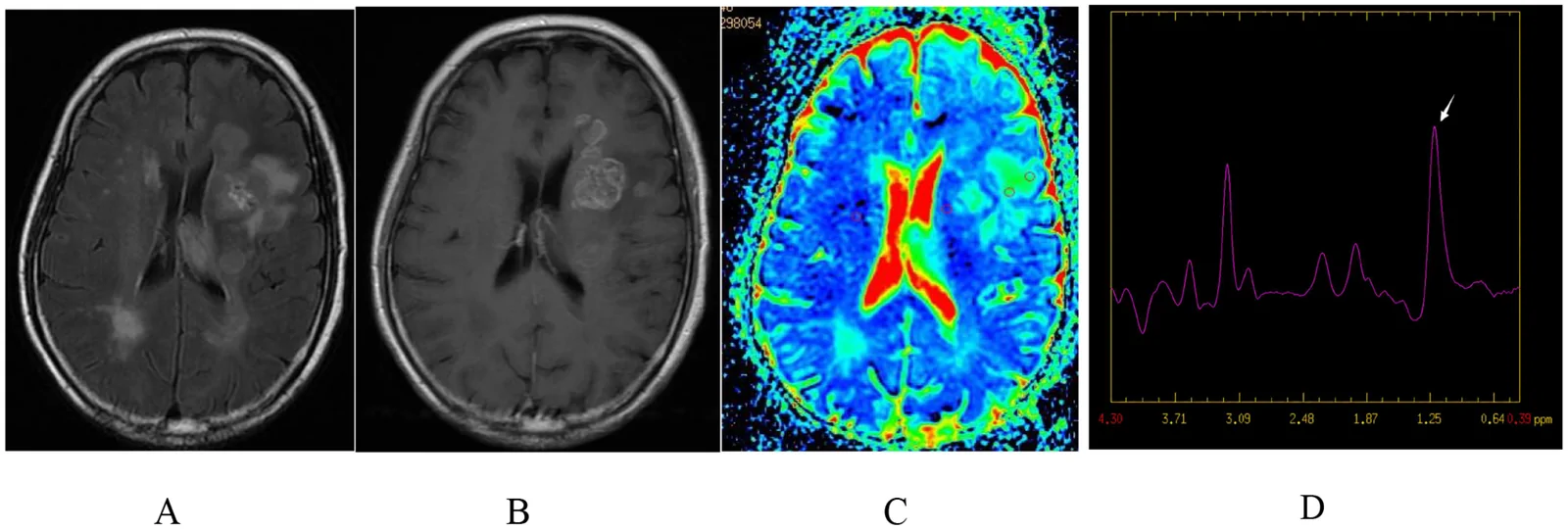
A 65-year-old man with atypical primary central nervous system lymphoma in the left corona radiata. **(A)** T2-FLAIR demonstrates heterogeneous hyperintensity and edema. **(B)** Contrast-enhanced T1-weighted imaging shows predominantly ring-like enhancement. **(C)** ADC map demonstrates marked diffusion restriction in viable tumor. **(D)** Proton MRS shows elevated choline, reduced N-acetylaspartate, and prominent lipid/lactate peaks.

## 4 Discussion

This study developed and internally validated a deliberately parsimonious model combining tumor ADC with Lip/Cr. Lower diffusivity and a higher lipid-region signal were independently associated with PCNSL. The combined model showed favorable corrected performance relative to tumor ADC alone; however, the direct comparison with Lip/Cr alone demonstrated only small, imprecise gains. The appropriate conclusion is therefore not that the combined model is superior, but that it is a plausible candidate for external validation whose incremental contribution beyond a carefully quality-controlled MRS marker remains uncertain.

The ADC difference is biologically plausible because the high cellular density and reduced extracellular space of PCNSL restrict water diffusion. Prior studies report lower tumor ADC in PCNSL than in glioblastoma (5–8). Nevertheless, necrosis, heterogeneous cellularity, partial-volume effects, echo-planar distortion, and ROI methodology produce overlap. Our point ROI approach targets viable low-ADC tumor and reports the mean across three adjacent sections, but the analytic file did not retain ROI area or whole-tumor measurements. Whole-tumor and fixed-area ROI sensitivity analyses should be incorporated prospectively rather than reconstructed from incomplete metadata.

Lip/Cr provided complementary metabolic information and was the strongest single predictor. Prominent lipid-region signals in PCNSL may reflect membrane turnover, microscopic necrosis, and the tumor microenvironment (9–11). However, TE 144 ms does not guarantee clean separation of lipid and lactate near 1.3 ppm, and automated workstation output remains sensitive to voxel placement, extracranial lipid contamination, water suppression, baseline stability, line width, and peak fitting. The retained dataset lacked numeric spectral quality metrics, repeated MRS measurements, and a candidate-level exclusion denominator. Consequently, the observed model performance must not be interpreted as evidence that Lip/Cr is equally reproducible across readers, scanners, or institutions.

The HGG group intentionally represented the pragmatic preoperative differential diagnosis but combined several WHO molecular entities (22). Restriction to IDH-wildtype glioblastoma yielded a corrected AUC of 0.891, which was directionally supportive but lower and imprecise. The small IDH-mutant astrocytoma and oligodendroglioma groups precluded reliable subtype-specific coefficients. This heterogeneity limits generalizability and should be addressed through larger stratified cohorts.

Proximal peritumoral ADC remained a secondary extension because that role was prespecified. Its estimated association and incremental changes were small and uncertain; interpretation therefore rests on effect magnitude and confidence intervals, not on P = 0.058. A geometric 0–10-mm peritumoral region does not represent a histologic infiltration boundary, and tract orientation, location, edema, and partial-volume effects further limit transportability (15).

The bootstrap procedure reduced but did not eliminate optimism. Repeating standardization within each resample prevented preprocessing leakage, and empirical calibration now shows local behavior rather than a line reconstructed from the intercept and slope. Nevertheless, the calibration-slope interval was wide. The log-Lip/Cr sensitivity analysis was stable, but all validation remained internal to one scanner and protocol. Differences in field strength, coil, b values, echo time, shimming, voxel placement, postprocessing, and disease spectrum can shift both ADC and MRS distributions; site-specific recalibration and harmonized quality control will therefore be necessary.

The apparent decision curve and threshold table illustrate possible trade-offs but do not establish clinical benefit. Prior reviews of machine-learning and radiomics differentiation studies also emphasize reporting limitations and risk of bias (18, 19). The present study did not compare radiologists alone with radiologists assisted by the model, define treatment-linked action thresholds, or prospectively evaluate changes in corticosteroid use, biopsy strategy, or resection planning. All such claims have been removed. Any future clinical role should be tested in an external reader-impact study in which the model provides a continuous probability as an adjunct to morphology and clinical context, while histopathology remains mandatory.

Several limitations remain. First, the availability-based cohort of 83 patients provides limited precision for calibration, non-linear effects, subtype analysis, and small performance increments. Second, the candidate-level screening log and the number of spectra rejected before analytic-file assembly were unavailable. Third, HGG was molecularly heterogeneous. Fourth, MRS reproducibility and numeric quality metrics were not retained. Fifth, ROI area and whole-tumor ADC were unavailable for sensitivity analysis. Sixth, the exploratory DCA used apparent predictions, and no prespecified clinical action threshold or prospective reader comparison was available. Finally, single-center internal validation cannot establish transportability. A multicenter external validation should harmonize acquisition, prospectively log exclusions, preregister MRS quality criteria, record ROI area, compare point and whole-tumor ADC, allow recalibration, and include a reader-impact analysis.

## 5 Conclusions

A two-parameter logistic model combining tumor ADC and Lip/Cr showed favorable exploratory performance on internal validation for differentiating PCNSL from HGG; however, its superiority over Lip/Cr alone was not established. Proximal peritumoral ADC added little and remained imprecise. The model is suitable for multicenter external validation with rigorous spectral and ROI quality control; it should not replace expert neuroradiologic assessment or histopathologic confirmation and should not independently determine treatment.

## Data Availability

The raw data supporting the conclusions of this article will be made available by the authors, without undue reservation.

## Supplementary material

Supplementary Figure 1 Distribution of Cho/Cr, Cho/NAA, and Lip/Cr by diagnosis. Horizontal lines indicate medians; points show individual observations. The distributions support use of median (interquartile range) and Mann–Whitney U tests for these metabolite ratios.
