# Supplementary material for: Tumor apparent diffusion coefficient combined with the lipid-to-creatine ratio for preoperative differentiation of primary central nervous system lymphoma from high-grade glioma: a single-center diagnostic model development and internal validation study
Source: Front Oncol. 2026 Sep 15;16:1941153. doi: 10.3389/fonc.2026.1941153 (PMC13619406; doi:10.3389/fonc.2026.1941153)
Supplement: Supplementary file 1 [file Image1.pdf]

Supplementary Figure

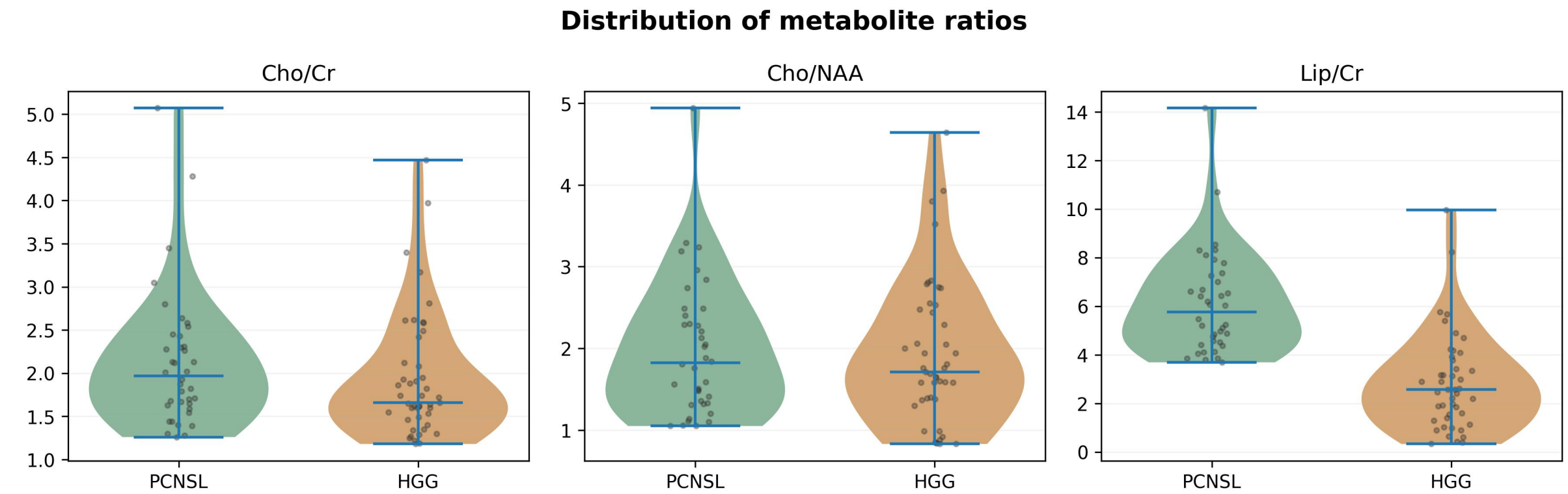

Supplementary Figure S1. Distribution of Cho/Cr, Cho/NAA, and Lip/Cr by diagnosis. Horizontal lines indicate medians; points show individual observations. The distributions support use of median (interquartile range) and Mann–Whitney U tests for these metabolite ratios.
